# Investigating functional fitness of older adults in Korea in the period 2013–2018

**DOI:** 10.1038/s41598-022-09969-1

**Published:** 2022-04-12

**Authors:** Bogja Jeoung, Do Young Pyun

**Affiliations:** 1 Gachon University, Incheon, Korea; 2 School of Sport, Exercise and Health Sciences, Loughborough University, Leicestershire, LE11 3TU UK

**Keywords:** Health policy, Public health

## Abstract

The purpose of this study is two-fold: (a) to examine differences in the body compositions and the functional fitness tests across genders, age groups, and the test periods; and (b) to develop the functional fitness standards for older Korean adults. This is a cross-sectional study using data from the Korea Institute of Sport Science Fitness Standards. The data were derived from 155,256 old adults aged 65–90 who participated in the National Fitness Award Project from 2013 to 2018 in Korea. A series of t-test, ANOVA, and Cajori’s 5-grade method were used to analyze the data. The group comparison tests revealed significant differences in the body compositions and the fitness tests across genders (*p* < 0.001 for all items), five age groups (*p* < 0.001 for all items), and two test periods (*p* < 0.001 for all items, except for sit-reach [*p* = 0.138] in males and body mass index [*p* = 0.258] and grip strength [*p* = 0.130] in females). The study also proposed the guidelines for the functional fitness standards for this population. This study would provide useful information to practitioners to design more effective physical activity programs or interventions for people at-risk of declining health, thus improving health conditions of the older adults in Korea.

## Introduction

The world’s aging population has been increasing at an unprecedented rate. The global population of 65 years and older reached 562 million (8% of total population) in 2012 and 617 million (8.5% of total population) in 2015^1^. It is predicted to reach 1.6 billion (17% of total population) by 2025^1^. Korea is no exception. Korea is getting ahead of the global trends and patterns in aging population. The population of 65 years and older in Korea reached 7% of its total population in 2000 and has been consistently increasing (e.g., 14.2% in 2017 and 15.7% in 2020)^2^. By 2025, the Korean population of 65 years and older is predicted to reach 20.3%, which is higher than the global expectation^2^. Furthermore, the life expectancy of Korean adults aged 65 years and over is 20.8 years (18.7 years for men, 22.8 years for women)^2^. This rate is also higher than the averages of other OECD countries (0.5 years for men and 1.5 years for women). Such an increase in the elderly population or population aging is known as one of the leading causes to a surge in healthcare costs because the annual healthcare expenditure for older adults is higher than that for other age groups^3^.

For example, cardiorespiratory fitness is known to be a powerful predictive factor that lowers the risk of mortality and morbidity, including the prevalence of metabolic syndrome, type 2 diabetes, and cardiovascular diseases^4,5^. Thus, many countries have focused on physical or other specific types of fitness to improve health of their citizens and reduce the social burden of medical care expenditure. The Korean government has been also trying to increase sport participation among people through the National Fitness Award Project (NFAP), which is a national testing and consultation service to promote functional fitness and health of all citizens. One study that examined how physical activities and fitness affected the relative risk of cardiovascular diseases showed that the relative risk of cardiovascular diseases decreased by 25% with physical activities and by 60% when physical fitness increased^6^. More interestingly, the study revealed maintaining physical fitness at satisfactory levels resulted in a dramatic decrease (40%) in relative risk of cardiovascular diseases even among people with physical fitness in the lower 25% group^6^. This infers that keeping good physical fitness and functional mobility in the elderly is important to improve their overall health. Several studies also supported that for older adults, improving and maintaining appropriate fitness not only improves the management of chronic diseases and decreases mortality, but also contributes to the capacity to accomplish everyday tasks, fall prevention, and improvement of cognitive function and quality of life^7–9^. For example, Berryman et al.^7^ revealed a positive association between physical fitness level and cognitive flexibility. In addition, Toraman and Yildirim^9^ highlighted that the enhancement of functional fitness performance such as upper and lower extremity muscle strength, aerobic endurance, agility, and dynamic balance significantly lowers a falling risk. Therefore, the maintenance of a high of appropriate level of functional fitness is important, particularly for older adults.

Functional fitness testing is a highly visible and important part of a fitness program. It is important for older adults to monitor fitness, evaluate health hazard-related factors and ensure safety, especially when engaging in new activities. Jones and Rikli^10^ developed and validated the physical fitness assessment instrument for older adults, grounded on a functional framework of physiological parameters required for basic and advanced daily activities. The researchers in Taiwan proposed physical fitness tests for older adults and emphasized several conditions required for these tests; these tests need to be effective, easy to perform, and require simple and inexpensive equipment^11^. Physical activities also need to be safe and enjoyable for older adults and, at the same time, meet the scientific standards for reliability and validity^11^. In a study of health-related fitness among Nepalese older adults^12^, five parameters were used to measure fitness, which were body compositions (e.g., BMI), hand grip strength, sit and reach, sit to stand, and the two-minute step test. Researchers in different countries have developed different parameters of fitness tests for older adults, according to their specific needs and circumstances.

In Korea, the NFAP for 65-year-olds and older individuals has been carried out since 2013. The fitness test parameters for older adults were developed on the consideration of several conditions such as relevance to basic and daily activity, inexpensive equipment, easy performance without risks of injuries, and the scientific standards with good reliability and validity. One of the most common methods to assess functional fitness is the use of a norm-referenced evaluation. In this way, older adults are able to monitor their own functional status and the relative position of an individual’s fitness performance level within a group^13^.

This study aimed to test if there were any differences in the functional fitness tests with regards to genders (males vs females), five age groups (65–69, 70–74, 75–79, 80–84, and 85–90), and two test periods (2013–2015 vs 2016–2018) for older adults in Korea, following the same fitness test battery and evaluation criteria on both genders and age groups, proposed by the NFAP. In addition, this study sought to develop the guidelines suggesting the standards for the functional fitness assessment norms across genders and the five age groups, using Cajori’s 5-grade evaluation method^14^. The findings of this study reflect the recent research trends on the functional fitness evaluations. By testing differences in various fitness abilities across gender, age groups, and different test periods, this study would provide useful information to practitioners to design more effective physical activity programs or interventions for people at-risk of declining health, thus improving health conditions of the older adults in Korea.

## Methods

### Research data

As a cross-sectional research, the data used for this study were drawn from the Korea Institute of Sport Science Fitness Standards as part of the NFAP, which were open access to the public. The NFAP has been implemented since 2013, and the Korea Sports Promotion Foundation released the six-year data (2013 to 2018) at the time of data collection. Nationally, there were 81 test centers across 17 regions in Korea. The participants in this study were aged 65–90 years. The data of the functional fitness tests from a total of 155,256 (51,751 males [33.3%] and 103,505 females [66.7%]) subjects who voluntarily participated at the centers from 2013 to 2018, along with their demographic information were used in this study.

### Functional fitness measurement

The functional fitness test battery for older adults was composed of seven components: (a) aerobic endurance (2-min step); (b) upper body muscle strength (hand grip strength); (c) lower body muscle endurance (chair sit and stand); (d) flexibility (sit and reach); (e) agility (timed up and go); and (g) body compositions (BMI and body fat). Height, weight, and blood pressure were also recorded. All parameters of functional fitness were measured at the designated centers on a voluntary basis. Each functional fitness test showed a high internal consistency, with satisfactory reliability statistics (r) ranging from 0.70 to 0.93^14^. The validity of the individual tests was not examined as this study used the publicly released data from the NFAP which were developed through the rigorous validation methods^13^ and have been successful implemented and utilized for the national fitness tests. The institute All test procedures were performed in accordance with the relevant guidelines and regulations and facilitated by certified national professional health and fitness instructors.

First, aerobic endurance was measured by the 2-min step test. The test began by customizing the minimum knee-stepping height and adjusting the level corresponding to a midway between the patella and the iliac crest. On the ‘go’ signal, the participants stepped in place as many times as possible over two minutes. The participants were instructed to step in place and raise their knees to a height halfway between the patella (kneecap) and the iliac crest (front hip bone). Second, for the measurement of upper body muscle strength, the hand grip strength test was used. This test was measured twice for left and right hands using a hand dynamometer, and a higher value was recorded to the nearest 0.1 kg. Third, to measure lower body muscle endurance, the chair sit and stand test was used. The participants were asked to stand up and sit down on the chair with both feet resting on the floor, and their hands were crossed at the wrists and held stationary on their shoulder. Subsequently, they performed the sitting down and standing up actions as many times as possible within 30 s. The number of stand-sit rounds was counted. Fourth, the sit and reach test was used to measure body flexibility. The participants were instructed to sit with bare feet, legs extended, toes pointed up, and feet approximately hip-wide apart, with the soles of the feet against the base of the measuring device. They were then asked to push the slide slowly forward, as far as they could, by placing one hand on top of the other, and without lifting their knees off the ground. Each participant performed the action twice, and a maximum height measurement was recorded to the nearest 0.1 cm. Fifth, the timed up and go test was used to measure agility. The participants started in a seated position on a standard chair with arm support. At the ‘go’ signal, they were instructed to stand up and walk as fast as possible to a pylon located 3 m in front of the chair. Without stopping their gait, the participants quickly turned around and came back to the chair and regained their initial seated position. The test was performed twice, and the results were expressed in 0.1 s. Finally, for body compositions, BMI was calculated by dividing body weight (kg) divided by height in meter squared (m^2^). The percent of body fat was measured using bioelectrical impedance analysis with a device called InBody, measuring the subjects’ height and blood pressure (systole and diastole in mmHg).

### Data analysis

As a prerequisite step, Mahalanobis distances were calculated to examine multivariate outliers using a critical value of chi-square at *p* = 0.001, and those cases deemed multivariate outliers were hence excluded from the data set. For the main analyses, first, a series of *t*-tests were used to determine if there were any differences in the body compositions and the fitness tests between genders at two different test periods (2013–2015 and 2016–2018), independently. Next, the comparisons across the five age groups (65–69, 70–74, 75–79, 80–84, and 85–90) for the body compositions and the fitness tests were conducted using one-way analysis of variance (ANOVA) at two different test periods (2013–2015 and 2016–2018). As a post-hoc test, the Scheffe test was carried out to find out which pairs of means were significant. Lastly, a series of *t*-tests were conducted to see if there were any significant differences in the body compositions and the fitness tests between 2013–2015 and 2016–2018 in both males and female groups, independently. The significance level was set at *p* < 0.05.

For the second purpose, Cajori’s (1928) 5-grade evaluation method were used to develop the functional fitness standards for the older adults with regards to their age groups and gender. The 5-grade relative evaluation was established: (a) excellent (7%, if M ≥  + 1.5σ); (b) very good (24%, if + 0.5σ < M <  + 1.5σ); (c) normal (38%, if − 0.5σ ≤ M ≤  + 0.5σ); (d) poor (24%, if − 0.5σ < M < −1.5σ); and (e) very poor (7%, if M ≤ − 1.5σ). The data were analyzed using SPSS version 21.0.

## Results

First, the study compared the body compositions and the fitness tests between males and females in each period of 2013–2015 and 2016–2018. For the average age, it was found that males were older than females in both periods. Regarding the body compositions, males were taller and heavier than females, but females recorded higher BMI and body fat in both periods. Males showed higher systole values in both periods. While there was no difference in diastole value in 2013–2015, males showed a higher diastole value in 2016–2018. Among the fitness tests, the values for grip strength, chair sit and stand, and 2-min step were higher for males who also showed better performance for timed up and go tests. However, the sit and reach scores were better for women. The same patterns of the findings, except diastole, were evidenced in both periods (see Table 1).

**Table 1:** Gender differences in functional fitness in 2013–2015 and 2016–2018.

	2013–2015						2016–2018					
	Male		Female		*t*	*p*	Male		Female		*t*	*p*
	N	M ± SD	N	M ± SD			N	M ± SD	N	M ± SD		
Age (year)	21,502	72.7 ± 5.1	45,051	72.1 ± 5.3	14.4	< 0.001	30,249	73.1 ± 5.4	58,454	72.4 ± 5.5	18.8	< 0.001
Height (cm)	21,502	165.0 ± 6.0	45,051	152.3 ± 5.5	258.8	< 0.001	30,249	165.1 ± 5.8	58,454	152.4 ± 5.5	313.1	< 0.001
Weight (kg)	21,502	65.8 ± 8.9	45,051	57.4 ± 7.9	117.2	< 0.001	30,249	66.2 ± 8.8	58,454	57.5 ± 7.9	143.9	< 0.001
Body mass index (kg/m^2^)	21,502	24.1 ± 2.7	45,051	24.7 ± 3.0	− 24.4	< 0.001	30,249	24.2 ± 2.7	58,454	24.7 ± 3.3	− 23.8	< 0.001
Body fat (%)	21,502	25.2 ± 8.8	45,051	34.6 ± 8.5	− 140.0	< 0.001	30,249	25.8 ± 6.5	58,454	34.8 ± 6.5	− 195.0	< 0.001
Systole (mmHg)	21,502	131.1 ± 15.0	45,051	128.7 ± 14.8	19.8	< 0.001	30,249	132.5 ± 14.7	58,454	130.0 ± 14.4	23.6	< 0.001
Diastole (mmHg)	21,502	76.5 ± 10.4	45,051	76.5 ± 9.8	− 0.4	.925	30,249	75.1 ± 10.1	58,454	74.8 ± 9.7	5.2	< 0.001
Grip strength (kg)	21,485	30.8 ± 6.9	44,923	19.2 ± 4.8	219.5	< 0.001	30,214	30.5 ± 6.6	58,365	19.3 ± 4.8	260.3	< 0.001
Sit and reach (cm)	21,499	4.0 ± 9.6	44,957	13.0 ± 7.9	− 119.0	< 0.001	30,155	3.9 ± 9.7	58,302	13.3 ± 7.9	− 144.0	< 0.001
Chair sit and stand (reps)	21,409	19.6 ± 7.1	44,778	17.5 ± 6.3	37.3	< 0.001	30,169	20.6 ± 6.4	58,184	18.4 ± 6.1	50.0	< 0.001
2-min step (reps)	19,886	105.4 ± 28.2	41,954	97.5 ± 30.0	31.3	< 0.001	28,789	107.9 ± 22.5	55,124	101.1 ± 25.6	39.8	< 0.001
Timed up and go (sec)	21,364	6.6 ± 2.9	44,733	7.2 ± 3.2	− 23.3	< 0.001	30,143	6.1 ± 1.7	58,285	6.7 ± 1.9	42.5	< 0.001

Second, an ANOVA test was performed to compare the body compositions and the fitness tests across the different age groups. Table 2 (2013–2016) and Table 3 (2016–2018) showed that a gradual decline in scores was observed over the five-year age spans for both males and females in height, weight, BMI, diastolic blood pressure, grip strength, sit and reach, chair sit and stand, and 2-min step. There was also a gradual decline with increasing ages in the performance of sit and reach, and timed up and go, for both males and females. The similar patterns of the results were observed in both test periods.

**Table 2:** Age differences in functional fitness for each gender (2013–2015).

		65–69	70–74	75–79	80–84	85–90	F	*p*
Height (cm)	Male	166.1 ± 5.8	165.2 ± 5.8	164.4 ± 5.9	162.5 ± 6.2	161.5 ± 6.1	193.1	< 0.001
	Female	154.0 ± 5.0	152.6 ± 5.2	150.8 ± 5.3	149.2 ± 5.4	147.8 ± 5.8	1263.4	< 0.001
Weight (kg)	Male	67.4 ± 8.6	66.3 ± 8.7	65.0 ± 8.8	62.1 ± 8.8	60.4 ± 8.7	202.3	< 0.001
	Female	58.5 ± 7.6	57.9 ± 7.7	56.6 ± 7.9	54.1 ± 8.2	51.7 ± 8.6	373.0	< 0.001
Body mass index (kg/m^2^)	Male	24.4 ± 2.6	24.2 ± 2.7	24.0 ± 2.8	23.4 ± 2.9	23.1 ± 2.9	64.3	< 0.001
	Female	24.6 ± 3.0	24.8 ± 3.0	24.8 ± 3.1	24.6 ± 3.2	23.8 ± 3.4	37.6	< 0.001
Body fat (%)	Male	24.1 ± 6.3	25.2 ± 7.4	25.9 ± 6.6	26.7 ± 10.7	27.6 ± 23.1	65.8	< 0.001
	Female	34.1 ± 7.9	34.8 ± 8.6	35.1 ± 7.4	35.1 ± 7.3	34.2 ± 7.7	34.7	< 0.001
BP systole (mmHg)	Male	129.8 ± 14.6	131.4 ± 14.9	132.0 ± 15.1	132.1 ± 15.9	131.6 ± 16.6	21.3	< 0.001
	Female	126.6 ± 14.3	128.8 ± 14.7	130.5 ± 14.9	131.8 ± 15.6	133.4 ± 15.7	191.8	< 0.001
BP diastole (mmHg)	Male	78.4 ± 9.8	76.8 ± 10.0	75.0 ± 10.4	73.8 ± 10.6	71.1 ± 11.0	146.5	< 0.001
	Female	77.4 ± 9.4	76.4 ± 9.7	75.7 ± 10.0	75.0 ± 10.5	75.9 ± 11.3	70.1	< 0.001
Grip strength (kg)	Male	33.4 ± 6.5	31.5 ± 6.3	28.9 ± 6.3	25.7 ± 6.4	23.8 ± 6.6	850.4	< 0.001
	Female	21.0 ± 4.3	19.5 ± 4.5	17.7 ± 4.7	15.9 ± 4.6	13.9 ± 4.7	1784.7	< 0.001
Sit and reach (cm)	Male	6.3 ± 9.2	4.3 ± 9.3	2.4 ± 9.6	0.22 ± 9.4	− 1.1 ± 9.7	251.9	< 0.001
	Female	15.1 ± 7.4	13.3 ± 7.6	11.2 ± 7.7	8.9 ± 7.8	6.4 ± 7.7	928.34	< 0.001
Chair sit and stand (reps)	Male	21.9 ± 6.4	20.0 ± 7.3	18.1 ± 6.1	15.8 ± 8.4	14.2 ± 5.0	467.4	< 0.001
	Female	19.5 ± 5.9	17.7 ± 6.1	15.6 ± 6.0	13.6 ± 5.6	11.5 ± 5.2	1377.5	< 0.001
2-min step (reps)	Male	113.7 ± 27.3	106.4 ± 26.8	100.3 ± 26.1	91.8 ± 33.2	83.3 ± 31.5	377.6	< 0.001
	Female	107.0 ± 25.7	99.7 ± 28.0	89.7 ± 31.1	78.2 ± 32.7	63.3 ± 31.8	1416.7	< 0.001
Timed up and go (s)	Male	5.9 ± 2.6	6.4 ± 2.5	7.0 ± 2.9	7.9 ± 3.0	8.6 ± 3.5	293.3	< 0.001
	Female	6.3 ± 2.1	7.0 ± 2.5	7.9 ± 3.2	9.2 ± 5.0	10.9 ± 6.7	1401.9	< 0.001

The Scheffe post-hoc tests revealed the significant differences on the following multiple comparisons:

BMI: All pairs, except 65–69 ≠ 70–74 and 80–84 ≠ 85–90 in males; 65–69 ≠ 80–84, 70–74 ≠ 75–79, 70–74 ≠ 80–84, 70–74 ≠ 85–90, 75–79 ≠ 80–84, and 80–84 ≠ 85–90 in females.

Body fat: All pairs, except 80–84 ≠ 85–90 in males; 65–69 ≠ 85–90, 70–74 ≠ 75–79, 70–74 ≠ 80–84, 70–74 ≠ 85–90, and 75–79 ≠ 80–84 in females.

BP systole: All pairs, except 65–69 ≠ 85–90, 70–74 ≠ 75–79, 70–74 ≠ 80–84, 70–74 ≠ 85–90, 75–79 ≠ 80–84, and 75–79 ≠ 85–90 in males.

BP diastole: All pairs, except 70–74 ≠ 85–90, 75–79 ≠ 85–90, and 80–84 ≠ 85–90 in females.

Grip strength: All pairs.

Sit and reach: All pairs, except 80–84 ≠ 85–90 in males.

Chair sit and stand: All pairs.

2-min step: All pairs.

Timed up and go: All pairs.

**Table 3:** Age differences in functional fitness for each gender (2016–2018).

		65–69	70–74	75–79	80–84	85–90	F	*p*
Height (cm)	Male	166.2 ± 5.7	165.3 ± 5.6	164.5 ± 5.7	163.4 ± 6.0	162.1 ± 6.3	220.1	< 0.001
	Female	153.9 ± 5.1	152.5 ± 5.2	151.0 ± 5.3	149.3 ± 5.5	147.3 ± 5.9	1513.7	< 0.001
Weight (kg)	Male	67.8 ± 8.6	66.7 ± 8.6	65.2 ± 8.6	63.6 ± 8.7	61.5 ± 9.5	234.5	< 0.001
	Female	58.4 ± 7.7	58.1 ± 7.7	56.8 ± 7.7	54.9 ± 8.2	52.4 ± 8.2	444.0	< 0.001
Body mass index (kg/m^2^)	Male	24.5 ± 2.6	24.3 ± 2.7	24.0 ± 2.7	23.7 ± 2.7	23.3 ± 3.1	77.8	< 0.001
	Female	24.6 ± 3.0	24.8 ± 3.8	24.9 ± 4.0	24.6 ± 3.3	24.1 ± 3.3	33.7	< 0.001
Body fat (%)	Male	24.9 ± 6.3	25.6 ± 6.1	26.3 ± 6.5	27.0 ± 6.5	27.5 ± 10.2	103.0	< 0.001
	Female	34.4 ± 6.4	35.0 ± 6.2	35.4 ± 6.5	35.2 ± 7.1	34.8 ± 7.5	56.4	< 0.001
Systole (mmHg)	Male	131.2 ± 14.4	132.5 ± 14.6	133.6 ± 15.0	133.7 ± 14.8	133.9 ± 15.1	34.7	< 0.001
	Female	128.0 ± 14.3	130.1 ± 14.4	131.9 ± 14.2	132.8 ± 14.4	133.6 ± 14.7	236.4	< 0.001
Diastole (mmHg)	Male	77.0 ± 9.7	75.4 ± 9.8	73.8 ± 10.2	73.0 ± 10.7	72.8 ± 10.8	160.7	< 0.001
	Female	75.49 ± 9.5	74.7 ± 9.6	74.4 ± 10.0	73.7 ± 10.3	73.4 ± 10.7	56.0	< 0.001
Grip strength (kg)	Male	32.9 ± 6.3	31.2 ± 6.1	29.1 ± 6.1	26.5 ± 6.1	23.9 ± 5.9	999.1	< 0.001
	Female	20.9 ± 4.2	19.7 ± 4.4	18.0 ± 4.6	16.1 ± 4.6	14.1 ± 4.7	2260.2	< 0.001
Sit-reach (cm)	Male	6.0 ± 9.3	4.5 ± 9.5	2.7 ± 9.6	0.08 ± 9.6	− 0.8 ± 9.7	320.0	< 0.001
	Female	15.1 ± 7.5	13.8 ± 7.7	11.8 ± 7.7	9.4 ± 7.8	7.4 ± 7.7	996.8	< 0.001
Chair sit-stand (reps)	Male	22.5 ± 6.2	21.3 ± 6.1	19.5 ± 6.1	17.1 ± 5.6	15.3 ± 5.3	706.4	< 0.001
	Female	20.2 ± 5.8	18.8 ± 5.8	16.8 ± 5.6	15.0 ± 5.1	13.0 ± 7.8	1565.0	< 0.001
2-min step (reps)	Male	114.3 ± 20.4	110.2 ± 20.8	104.8 ± 21.9	96.1 ± 24.4	88.8 ± 26.7	620.4	< 0.001
	Female	109.1 ± 21.1	103.7 ± 23.1	94.4 ± 25.8	85.0 ± 30.6	75.2 ± 28.6	1852.2	< 0.001
Timed up and go (s)	Male	5.6 ± 1.4	5.9 ± 1.4	6.4 ± 1.7	7.2 ± 1.9	8.1 ± 2.5	969.6	< 0.001
	Female	5.9 ± 1.2	6.4 ± 1.5	7.3 ± 2.0	8.3 ± 2.5	9.4 ± 3.2	3506.0	< 0.001

The Scheffe post-hoc tests revealed non-significant differences on the following multiple comparisons:

BMI: 65–69 ≠ 80–85 and 70–74 ≠ 75–79 in females.

Body fat: 80–84 ≠ 85–90 in males; 65–69 ≠ 85–90, 70–74 ≠ 80–84, 70–74 ≠ 85–90, 75–79 ≠ 80–84 in females.

BP systole: 70–74 ≠ 85–90, 75–79 ≠ 80–84, 75–79 ≠ 85–90, 80–84 ≠ 85–90 in males; 80–84 ≠ 85–90 in female.

BP diastole: 75–79 ≠ 85–90; 80–84 ≠ 85–90 in males; 70–74 ≠ 75–79, 80–84 ≠ 85–90 in females.

Sit and reach: 80–84 ≠ 85–90 in males.

Third, the body compositions and the fitness tests between 2013–2015 and 2016–2018 were compared in both male and female groups and the results were reported in Table 4. In the male groups, weight and body fat scores were higher in 2016–2018. The systolic blood pressure was also higher in 2016–2018. However, the diastolic blood pressure was lower in 2016–2018. Moreover, the height, weight, and BMI showed no difference among women in 2013–2015 versus 2016–2018. However, in the female group, body fat and systolic blood pressure were higher in 2016–2018, and the diastolic blood pressure was higher in 2013–2015. Comparing the fitness tests between two periods (2016–2018 and 2013–2015), the male participants showed better performance in grip strength, chair sit and stand, 2-min step, 6-min walk, and timed up and go in 2016–2018. The female participants showed better performance in all parameters in 2016–2018.

**Table 4:** Test period differences in functional fitness for each gender.

	Male				Female			
	2013–2015	2016–2018	*t*	*p*	2013–2015	2016–2018	*t*	*P*
Age (year)	72.7 ± 5.2	73.1 ± 5.4	− 7.7	< 0.001	72.1 ± 5.5	72.4 ± 5.5	− 8.3	< 0.001
Height (cm)	165.0 ± 6.0	165.1 ± 5.8	− 1.2	0.238	152.5 ± 5.5	152.4 ± 5.5	0.118	0.783
Weight (kg)	65.8 ± 8.9	66.2 ± 8.8	− 4.1	< 0.001	57.4 ± 7.9	57.5 ± 7.9	− 0.373	0.495
Body mass index (kg/m^2^)	24.1 ± 2.7	24.2 ± 2.7	− 4.0	< 0.001	24.7 ± 3.0	24.7 ± 3.3	− 1.0	0.258
Body fat (%)	25.2 ± 8.0	25.8 ± 6.5	− 8.0	< 0.001	34.6 ± 8.0	34.8 ± 6.5	− 5.3	< 0.001
Systole (mmHg)	131.1 ± 15.0	132.5 ± 14.7	− 10.3	< 0.001	128.7 ± 14.8	130.0 ± 14.4	− 15.0	< 0.001
Diastole (mmHg)	76.5 ± 10.2	75.1 ± 10.1	14.6	< 0.001	76.5 ± 9.8	74.8 ± 9.7	27.8	< 0.001
Grip strength (kg)	30.8 ± 6.9	30.5 ± 6.6	5.0	< 0.001	19.2 ± 4.8	19.3 ± 4.8	− 0.94	0.130
Sit-reach (cm)	4.0 ± 9.6	3.9 ± 9.7	1.3	0.138	13.0 ± 7.9	13.3 ± 7.9	− 4.8	< 0.001
Chair sit-stand (reps)	19.6 ± 7.1	20.6 ± 6.4	− 15.6	< 0.001	17.5 ± 6.3	18.4 ± 6.1	− 22.0	< 0.001
2-min step (reps)	105.4 ± 28.2	107.4 ± 22.5	− 10.4	< 0.001	97.5 ± 30.4	101.1 ± 25.6	− 18.7	< 0.001
Timed up and go (sec)	6.6 ± 2.9	6.1 ± 1.7	20.8	< 0.001	7.2 ± 3.2	6.7 ± 1.9	28.0	< 0.001

Lastly, the functional fitness components by age and gender, as developed using Cajori’s 5-grade evaluation standards, are presented in Table 5. The results revealed a consistent pattern of decline in performance in all test variables, for both men and women, over the 5-year age categories.

**Table 5:** Functional fitness 5-grade relative evaluation standards.

	Gender	Age group	Very poor (7%)	Poor (24%)	Good (38%)	Very good (24%)	Excellent (7%)
Grip strength	Male	65–69	≤ 23.5	23.6 to 29.0	29.1 to 36.3	36.4–42.2	42.3 ≤
		70–74	≤ 22.0	22.1 to 27.3	27.4 to 34.5	34.6–40.0	40.1 ≤
		75–79	≤ 19.7	19.8 to 25.1	25.2 to 32.1	32.2–37.9	38.0 ≤
		80–84	≤ 16.3	16.4 to 22.1	22.2 to 29.4	29.5–34.0	34.1 ≤
		85–90	≤ 13.5	13.6 to 19.6	19.7 to 27.3	27.4–32.5	32.6 ≤
	Female	65–69	≤ 14.7	14.8 to 18.1	18.2 to 23.0	23.1–27.0	27.1 ≤
		70–74	≤ 12.9	13.0 to 16.7	16.8 to 21.8	21.9–25.8	25.9 ≤
		75–79	≤ 10.8	10.9 to 14.8	14.9 to 20.2	20.3–24.3	24.4 ≤
		80–84	≤ 9.2	9.3 to 12.7	12.8 to 18.4	18.5–22.6	22.7 ≤
		85–90	≤ 7.1	7.2 to 10.5	10.6 to 16.3	16.4–21.2	21.3 ≤
Chair sit and stand	Male	65–69	≤ 14	15 to 18	19 to 25	26–32	33 ≤
		70–74	≤ 12	13 to 16	17 to 24	25–30	31 ≤
		75–79	≤ 11	12 to 15	16 to 22	23–28	29 ≤
		80–84	≤ 9	10 to 13	14 to 19	20–26	27 ≤
		85–90	≤ 8	9 to 11	12 to 17	18–23	24 ≤
	Female	65–69	≤ 12	13 to 16	17 to 22	23–29	30 ≤
		70–74	≤ 11	12 to 14	15 to 21	22–28	29 ≤
		75–79	≤ 9	10 to 12	13 to 19	20–25	26 ≤
		80–84	≤ 8	9 to 11	12 to 17	18–22	23 ≤
		85–90	≤ 5	6 to 9	10 to 14	15–20	21 ≤
2-min step	Male	65–69	≤ 85	86 to 102	103 to 123	124–144	145 ≤
		70–74	≤ 78	79 to 97	98 to 119	120–139	140 ≤
		75–79	≤ 69	70 to 91	92 to 114	115–133	134 ≤
		80–84	≤ 50	51 to 80	81 to 108	109–127	128 ≤
		85–90	≤ 40	41 to 68	69 to 102	103–122	123 ≤
	Female	65–69	≤ 76	77 to 96	97 to 119	120–138	139 ≤
		70–74	≤ 64	65 to 89	90 to 114	115–134	135 ≤
		75–79	≤ 47	48 to 78	79 to 107	108–127	128 ≤
		80–84	≤ 31	32 to 63	64 to 99	100–120	121 ≤
		85–90	≤ 20	21 to 48	49 to 88	89–111	112 ≤
Sit and reach	Male	65–69	≤ − 8.9	− 8.8 to 0.1	0.2 to 11.2	11.3–19.1	19.2 ≤
		70–74	≤ − 10.0	− 10.1 to − 1.7	− 1.6 to 9.3	9.4–17.7	17.8 ≤
		75–79	≤ − 12.0	− 11.9 to − 4.0	− 3.9 to 7.6	7.7–16.1	16.2 ≤
		80–84	≤ − 15.0	− 14.9 to − 7.0	− 6.9 to 5.0	5.1–14.0	14.1 ≤
		85–90	≤ − 16.0	− 15.9 to − 8.3	− 8.2 to 4.5	4.6–12.2	12.3 ≤
	Female	65–69	≤ 3.4	3.5 to 10.3	10.4 to 19.3	19.4–25.0	25.1 ≤
		70–74	≤ 2.0	2.1 to 8.6	8.7 to 17.9	18.0–23.9	24.0 ≤
		75–79	≤ 0.0	0.1 to 6.7	6.8 to 15.8	15.9–22.0	22.1 ≤
		80–84	≤ − 2.8	− 2.7 to 4.2	4.3 to 13.5	13.6–20.0	20.1 ≤
		85–90	≤ − 5.0	− 4.9 to 2.0	2.1 to 11.0	11.1–17.5	17.6 ≤
Timed up and go	Male	65–69	≥ 7.7	7.6 to 6.1	6.0 to 4.9	4.8–4.4	4.3 ≥
		70–74	≥ 8.3	8.2 to 6.5	6.4 to 5.2	5.1–4.6	4.5 ≥
		75–79	≥ 9.2	9.1 to 7.0	6.9 to 5.5	5.4–4.8	4.7 ≥
		80–84	≥ 10.9	10.8 to 8.1	8.0 to 6.1	6.0–5.3	5.2 ≥
		85–90	≥ 12.3	12.2 to 8.8	8.7 to 6.5	6.4–5.7	5.6 ≥
	Female	65–69	≥ 8.0	7.9 to 6.5	6.4 to 5.3	5.2–4.7	4.6 ≥
		70–74	≥ 9.1	9.0 to 7.1	7.0 to 5.6	5.5–5.0	4.9 ≥
		75–79	≥ 10.7	10.6 to 8.0	7.9 to 6.2	6.1–5.4	5.3 ≥
		80–84	≥ 12.6	12.5 to 9.2	9.1 to 6.9	6.8–5.9	5.8 ≥
		85–90	≥ 15.0	14.9 to 10.5	10.4 to 7.6	7.5–6.3	6.2 ≥

## Discussion

The quality of life of older adults depends on the ability to continue doing what they want without physical pains as long as possible. The important goals of maintaining older adults’ fitness are to prevent physical frailty and improve functional mobility. Functional fitness testing is as important for older adults as it is for other age groups. The results can be used to guide the basis and management of chronic diseases, highlight the achievable daily activities, and contribute to the design of a suitable exercise program for general health and fitness purposes. The current study provides the fitness data for older adults (aged from 65 to 90) at three-year intervals from 2013 to 2018, where the participants were categorized into the five-year age groups. We used the raw data from the NFAP which were collected at the national fitness centers located in 17 regions of Korea. This is the first attempt to develop the functional fitness standards for older Korean adults, according to Cajori’s five grades, ranging from very poor to excellent.

The NFAP’s functional fitness assessment criteria for older adults (65 +) are presented with three stages: gold, silver, and bronze medals. Gold model is achieved when all testing components are at least 70th percentile (above 30%), followed silver medal with above 50th percentile and bronze medal with above 30th percentile (below 30%)^15^. Instead, the current study proposes the functional fitness assessment criteria over five stages: excellent (7%)—very good (24%)—good (38%)—poor (24%)—very poor (7%). Compared to the NFAP’s gold medal (30%), our findings from the ‘excellent-very good’ assessment criteria (31%) showed lower scores in grip strength for all gender and age groups. However, in the same assessment criteria, our findings revealed higher scores in the remaining tests for all gender and age groups. It indicates the NFAP’s fitness assessment criteria fail to reflect the fitness conditions of the current older adults in Korea. Hence, the new guidelines for the functional fitness assessment would provide more accurate information for practitioners to develop new policies and programme for this population.

For the measurement of aerobic endurance, the NFAP has implemented both 2-min step and 6-min walk as they are considered effective tools to measure aerobic endurance which is highly associated with physical performance as well as the cardiovascular and respiratory systems of older adults. At the fitness centers, the participants had an opportunity to choose either test for aerobic endurance testing, and 92% of them selected 2-min step. In this study, accordingly, we used the data from 2-min step to analyze aerobic endurance. In the research by Chen et al.^11^, most Taiwanese older adult participants (83%) chose the 2-min step test over the 3-min step test using the step box for aerobic endurance testing. As such, Chen et al.^11^ recommended removing the 3-min step test from the aerobic endurance assessment. The aerobic endurance test has been mainly used in past studies related to health and functional fitness^12,16^. A unified examination may be necessary to produce more reliable outcomes for aerobic endurance across participants.

Significantly higher values were found in 2015–2018 than in 2013–2015, regardless of gender and across all specific age groups (65–69, 70–74, 75–79, 80–84, 85–90). Compared to other studies^10–12,17,18^, the aerobic endurance scores in terms of genders and different age groups seem higher than those in these studies. The evaluation standard scores found in our study also seem higher than those of other studies with regards to gender and age^10–12^.

Functional fitness parameters of motor ability in older adults usually refer to power, speed/agility, and balance. In this study, timed up and go was used to assess for agility which involves getting up from a seated position and walking as quickly as possible to a certain distance while keeping balance and then returning to the original seated position. These movements help reduce an individual’s risk of falling and mobility problems. In addition, recent research showed that a human brain has the ability to control the gait and movement of older adults and confirmed an association between motor ability and brain^19,20^. Past researchers found that it is hard to predict motor ability once certain signs of aging of brain surface^20,21^. For motor ability assessments, timed up and go has been popularly utilized to predict the aging of brain.

Older adults are at high risk of falling. The proportion of Korean older adults over 65 years of age with fall experience is 62.8%^21^. Falling is a factor that is highly related to lower body muscles, balance, agility, and fitness. We believe that participation in physical activities should increase and that a proposal for a standard for fall prevention through each individual’s motor ability assessment should be developed to provide guidelines that the general public can understand and provide appropriate prescriptions for each individual.

This study showed that men in all age groups scored higher for all parameters. The analysis by age group showed a gradual decline in these parameters in both men and women, consistent with results of existing studies^10,12,14,18^. This study also demonstrated that both men and women scored higher for these parameters in 2015–2018 than in 2013–2015, across all the specific age groups. In the study, muscle strength and muscular endurance were assessed using grip strength and chair sit and stand, respectively. Flexibility was assessed with sit and reach. These parameters showed higher scores in men of all ages than women. A gradual decline was observed in each age group of both genders, similar to previous studies^10,12,17,18^. Moreover, the analysis of these parameters showed higher scores in 2016–2018 than 2013–2015 in both genders and across all age groups; the scores in 2013–2015 and 2016–2018 for Korean older adults were higher than those for older adults in other countries^10,12,17^.

A reduction in lean body mass and an increase in fat body mass can increase the mortality risk of older adults. Therefore, it is essential to maintain a healthy body shape that has more muscle mass than fat body mass. As aging proceeds, fat and lean soft tissues tend to change due to a sedentary lifestyle and malnutrition. In this research, both men and women showed a tendency for a decreased BMI and body fat % as age increased. Older women were exposed to a higher hazard ratio because they showed higher body fat %, an indicator of obesity. BMI and body fat % slightly increased from 2013–2015 to 2016–2018 for both men and women, and these were reflected in the increased hazard ratio for Korean older adults. The World Health Organization recommends a BMI range of 23–28 kg/m^2^ for older adults, and the Asia–Pacific region and the Korean Society for the Study of Obesity recommend a BMI range of 23–25 kg/m^2^.

Even in the assessment of physique, height and weight for both men and women tended to decrease as age increased, and height and weight had increased from 2013 to 2015 to 2016–2018. In the NFAP testing facilities, blood pressure was measured in addition to performing the fitness tests. The hypertension guidelines published by the American Heart Association and American College of Cardiology propose the normal levels for systole/diastole to be under 120/80 mmHg and the stage 1 hypertension level to be > 130/80 mmHg. However, the Korean Academy of Medical Science and Korean Disease Control and Prevention Agency proposes blood pressure levels of 120–129 mmHg as elevated and > 140/90 mmHg as stage 1 hypertension^22^. Older men were shown to be pre-hypertensive while older women had elevated blood pressure. Korean older adults are in the cardio-cerebrovascular disease risk group, and their blood pressure increased with increase in age^23^.

Compared to physical fitness conditions of the Nepalese older adults^12^, the Korean older adults showed better fitness performance in all fitness tests while they reported higher scores in BMI and Body fat. When compared to the Portuguese functional fitness normative scores suggested by Rikli and Jones^24^, the current results in the criteria of excellent (7%) and very good (31%) showed better performance for all gender and age groups. Moreover, the Korean older adults reported higher scores for all functional tests than Portuguese^25^ and Spanish^26^ older adults. One possible reason explaining a good functional fitness among Korean older adults is that the Korean government has regularly examined their fitness conditions and provided customized exercise programs for free of charge which result in active participation in physical activities.

While Korean older adults scored higher for functional fitness than older adults in Western or other Asian countries, they are at risk of cardio-cerebrovascular diseases because their BMI, body fat %, and blood pressure were higher than the standard norms. Based on the findings of this study, older adults should be encouraged to participate in nutritional management, physical activities, and exercise programs to manage their obesity and also be provided with education to change their awareness. Furthermore, the NFAP testing facilities should regularly update fitness criteria every two or three years by monitoring changes of functional fitness among older adults, provide accurate criteria for the health assessments that facilitate the accurate monitoring of the functional health conditions of older adults, and encourage participation in physical activities.

## Conclusions

By analysing data derived from the functional fitness assessments for older adults, we were able to understand the changes in physical fitness of older adults and their health conditions. Our findings could support related policymakers or practitioners in monitoring the physical health conditions of older adults and further help develop appropriate activities and exercise programs for this selected population. Regular sport and exercise participation among older adults would contribute to more healthy lifestyles in future years. To enhance our current knowledge of physical fitness in an aging population, further research on a cross-disciplinary approaches (physical activities, urban studies, gerontology, cardiology, etc.) and health-related longitudinal studies need to be continued.

## Data Availability

The data used for this research can be downloaded for free from the website of Korea Sports Promotion Foundation: https://www.bigdata-culture.kr/bigdata/user/data_market/detail.do?id=ace0aea7-5eee-48b9-b616-637365d665c1.
